# Supplementary material for: Lantern-shaped flexible RNA origami for Smad4 mRNA delivery and growth suppression of colorectal cancer
Source: Nat Commun. 2023 Mar 10;14:1307. doi: 10.1038/s41467-023-37020-y (PMC9998469; doi:10.1038/s41467-023-37020-y)
Supplement: Supplementary file 1 — Supporting Information [file 41467_2023_37020_MOESM1_ESM.pdf]

Lantern-shaped flexible RNA origami for *Smad4* mRNA delivery and growth suppression of colorectal cancer

Muren Hu[1, 2, #], Chang Feng[3, #], Qianqin Yuan[1, #], Chenbin Liu[1], Bujun Ge[2,*], Fenyong Sun[1,*], Xiaoli Zhu[1,*]

[1]Department of Clinical Laboratory Medicine, Shanghai Tenth People's Hospital, School of Medicine, Tongji University, Shanghai, 200072, P. R. China.
[2]Department of General Surgery, Tongji Hospital, School of Medicine, Tongji University, Shanghai, 200065, P. R. China.
[3]Center for Molecular Recognition and Biosensing, School of Life Sciences, Shanghai University, Shanghai, 200444, P. R. China.
[#]These authors contributed equally to this work.
[*]Corresponding Authors:
Bujun Ge, E-mail: gebujun@126.com.
Fenyong Sun, E-mail: sunfenyong@263.net.
Xiaoli Zhu, E-mail: xiaolizhu@shu.edu.cn.

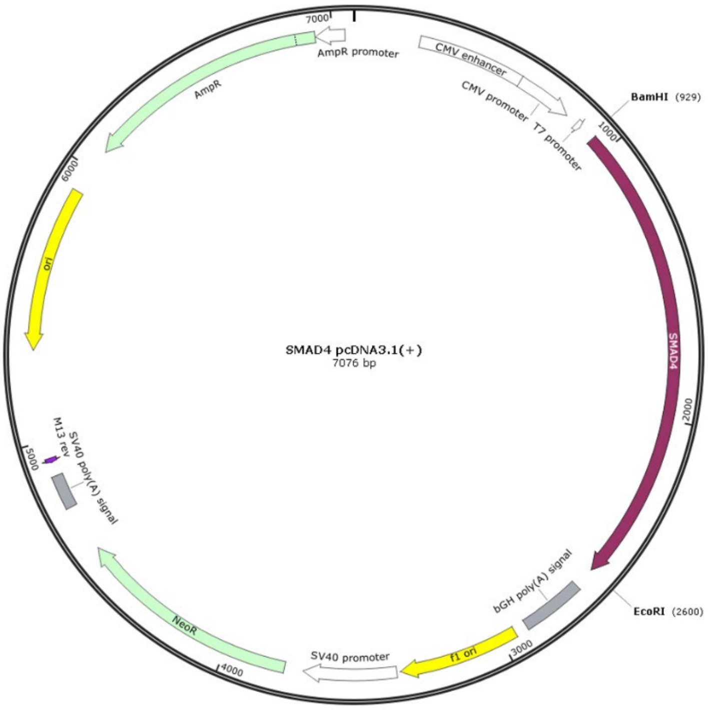

Supplementary Figure 1. pCDNA 3.1-*Smad4* vector containg T7 promoter.

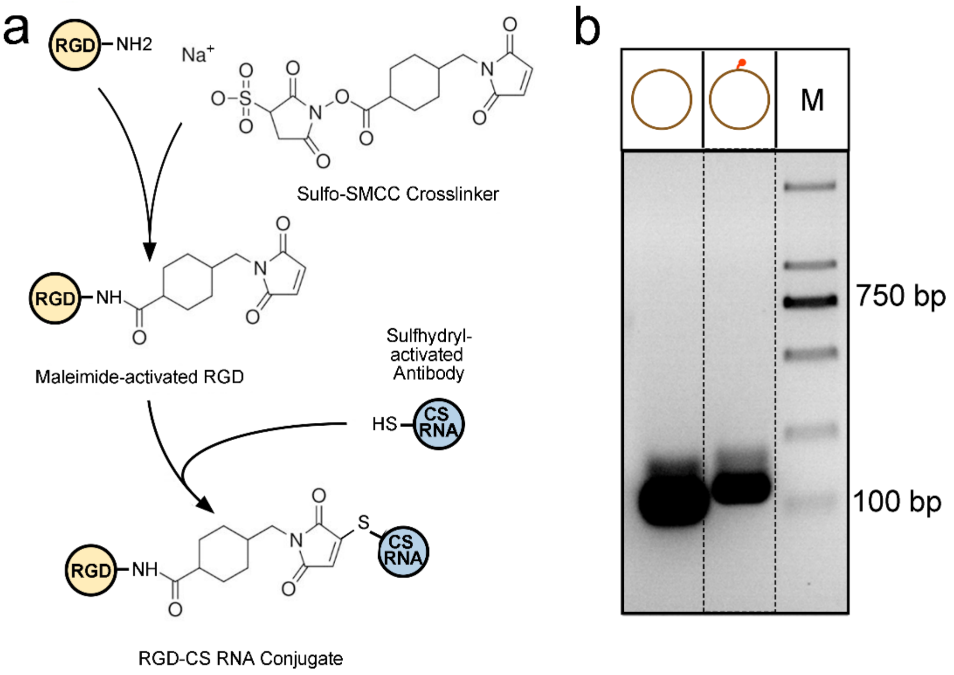

Supplementary Figure 2. Synthesis of RGD-CS RNA conjugates. (a) Synthetic pathway for RGD and oligonucleotide conjugation. (b) Representative agarose gel electrophoresis characterization of the RGD-CS RNA conjugates from 3 independent experiments. Source data are provided as Source Data file.

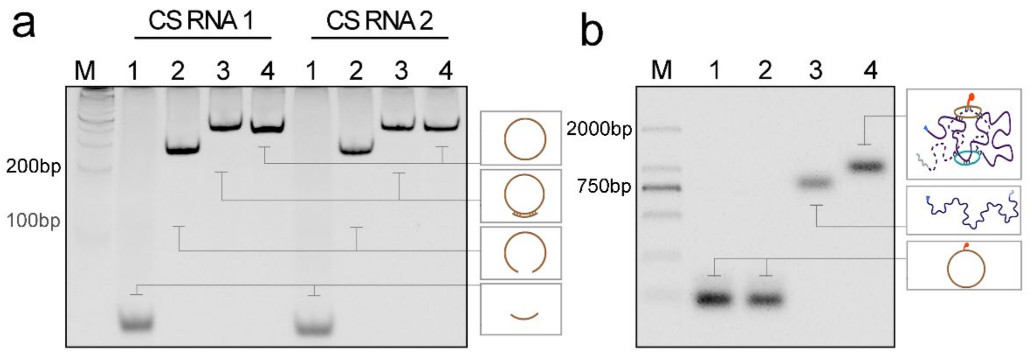

Supplementary Figure 3. Representative urea-polyacrylamide gel electrophoresis of circular RNA staples (3-bs) synthesize (a) and agarose gel electrophoresis of the formation of *Smad4* mRNA nano-lantern (b) from 3 independent experiments, created with BioRender.com. Source data are provided as Source Data file.

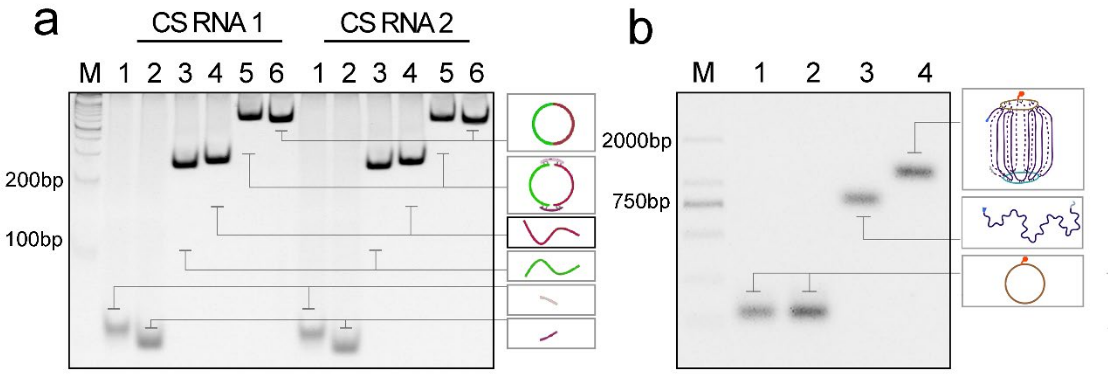

Supplementary Figure 4. Representative urea-polyacrylamide gel electrophoresis of circular RNA staples (7 bs) synthesize (a) and agarose gel electrophoresis of the formation of *Smad4* mRNA nano-lantern (b) from 3 independent experiments, created with BioRender.com. Source data are provided as Source Data file.

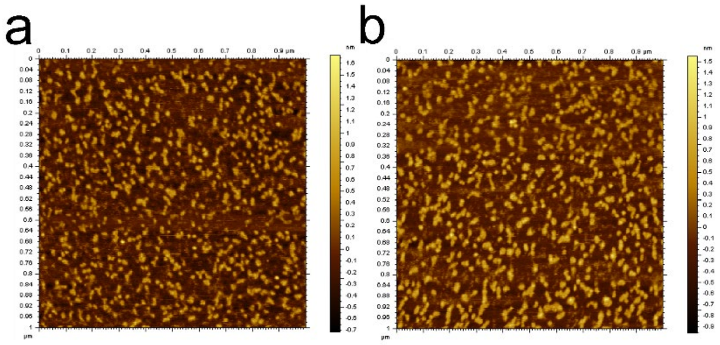

Supplementary Figure 5. Representative AFM characterization of RNA staple couples with different numbers of binding sites, 3-bs (a), 7-bs (b), from 3 independent experiments.

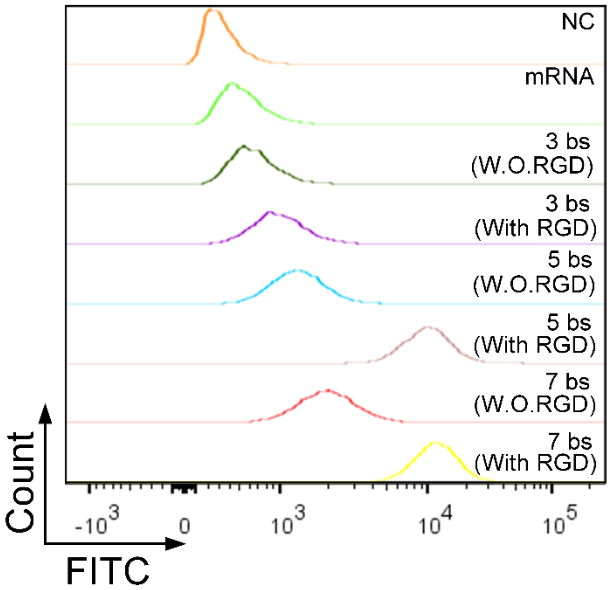

Supplementary Figure 6. Intake profiles of FITC labeled nano-lantern (with 3, 5, and 7 bs) with or without RGD in SW480 cell.

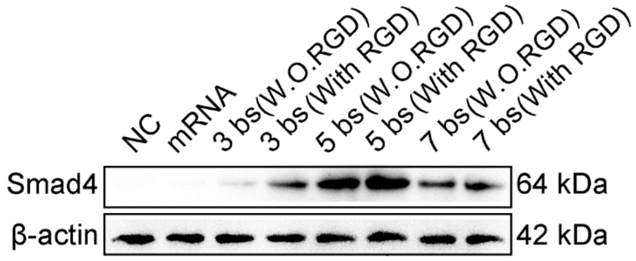

Supplementary Figure 7. Representative western blot analysis of intracellular Smad4 expression in SW480 cells after transfection with 3 bs, 5 bs and 7 bs nano-lanterns with or without RGD from 3 independent experiments. Source data are provided as Source Data file.

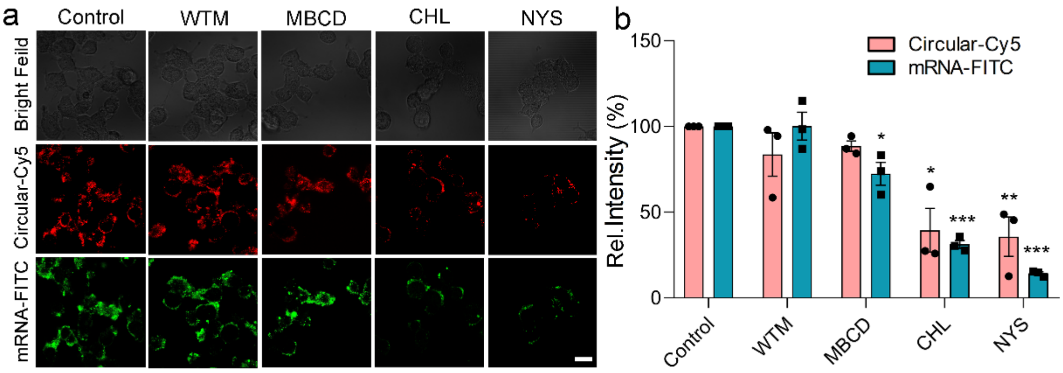

Supplementary Figure 8. Investigation of cellular uptake mechanism. (a) Confocal images of SW480 cells after treated with nano-lanterns and different inhibitors. The inhibitors are as follows: wortmannin (WTM, inhibitor of macropinocytosis), methyl-beta-cyclodextrin (MBCD, inhibitor of cholesterol-dependent endocytosis), chlorpromazine (CHL, inhibitor of clathrin-mediated endocytosis), and nystatin (NYS, inhibitor of lipid raft-caveolae endocytosis). Scale bar: 20 μm. (b) Statistical results of the relative fluorescence intensities of Cy5 and FITC obtained from (a), n = 3 biologically independent experiments. Data are presented as the mean ± SEM, Statistical differences were assessed using one-way ANOVA with Bonferroni multiple comparisons test. Circular-Cy5 (Control vs. CHL, p = 0.012; Control vs. NYS, p = 0.008), mRNA-FITC (Control vs. MBCD, p = 0.024; Control vs. CHL, p < 0.001; Control vs. NYS, p < 0.001). *, p<0.05, **, p<0.01, ***, p<0.001. Source data are provided as Source Data file.

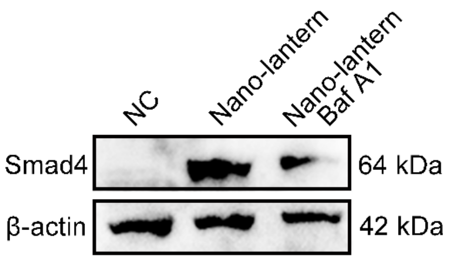

Supplementary Figure 9. Representative western blot analysis of Smad4 expression in SW480 cells after treatment with nano-lantern and Baf A1 from 3 independent experiments. The cells were pre-incubated for 30 min in serum-free medium containing Baf A1 (200 nM) inhibitors for intracellular proton-pump effects. Source data are provided as Source Data file.

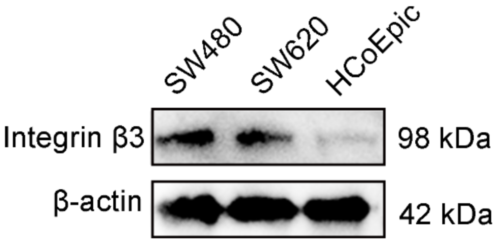

Supplementary Figure 10. Representative western blot analysis the Integrin β3 expression in SW480, SW620, and HcoEpic cell lines from 3 independent experiments. Source data are provided as Source Data file.

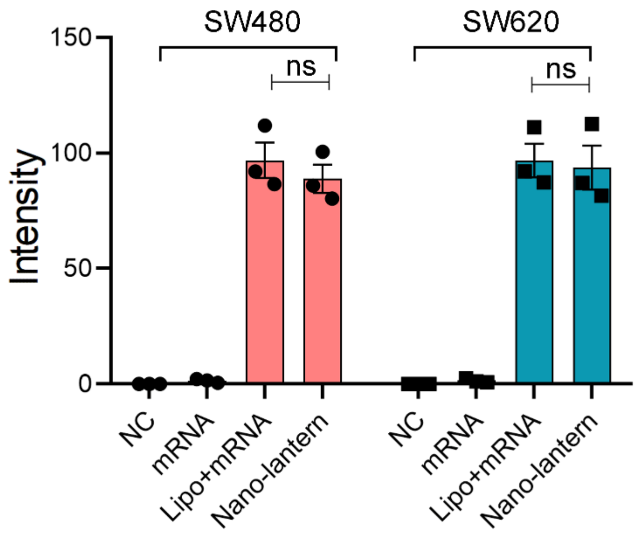

Supplementary Figure 11. Intensity statistical of Smad4 protein expression in SW480 and SW620 cells treated with individual *Smad4* mRNA, Lipo+mRNA, and nano-lantern, Data are presented as the mean ± SEM. n=3 independent biological samples. Statistical differences were assessed using one-way ANOVA with Bonferroni multiple comparisons test. ns, nonstatistically significant. Source data are provided as Source Data file.

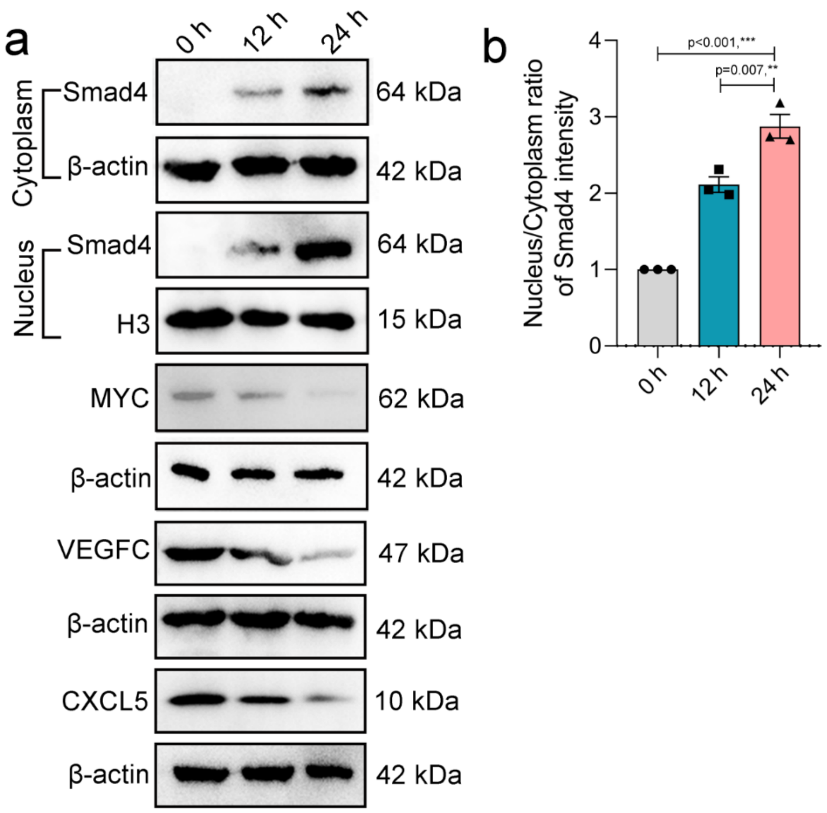

Supplementary Figure 12. Nuclear translocation of Smad4 in SW480 cell lines after transfection with nano-lantern. (a) Western blot analysis of Smad4 expression in nucleus and cytoplasm and its downstream genes' expression. (b) Intensity statistical of the nucleus/cytoplasm ratio of Smad4 expression from panel a, n=3 biologically independent experiments. Data are presented as the mean ± SEM. Statistical differences were assessed using one-way ANOVA with Bonferroni multiple comparisons test. *, $p<0.05$, **, $p<0.01$, ***, $p<0.001$. Source data are provided as Source Data file.

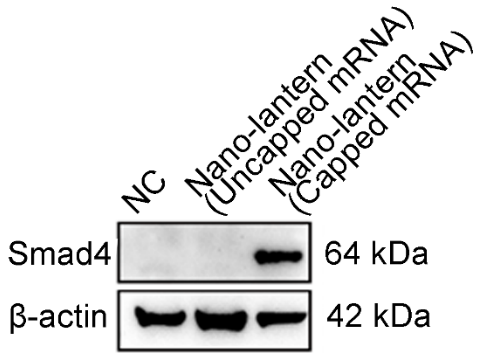

Supplementary Figure 13. Representative western blot analysis of Smad4 expression in SW480 cells after treatment with nano-lantern (Uncapped mRNA), nano-lantern (Capped mRNA) from 3 independent experiments. Source data are provided as Source Data file.

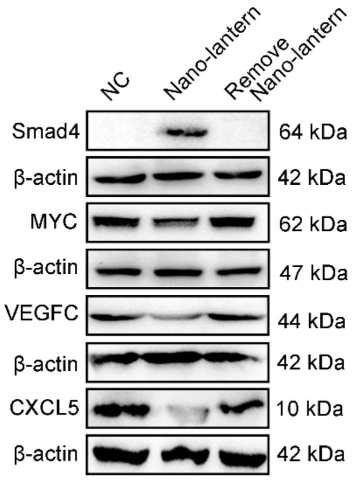

Supplementary Figure 14. Representative western blot analysis of Smad4, MYC, VEGFC and CXCL5 expression in SW480 cells after removing nano-lantern from 3 independent experiments. Source data are provided as Source Data file.

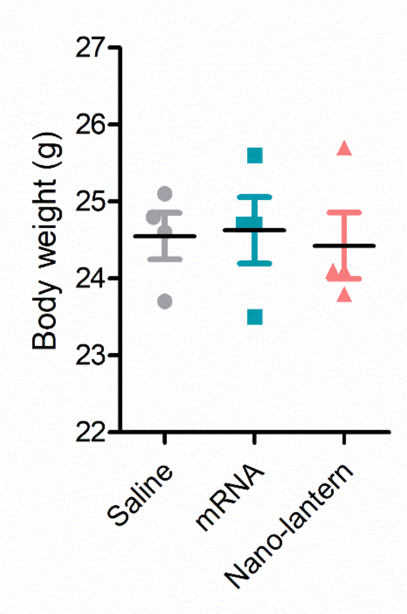

Supplementary Figure 15. Body weight of orthotopic tumor bearing mice with different treatments, n=4 mice/group, data are presented as the mean ± SEM. Source data are provided as Source Data file.

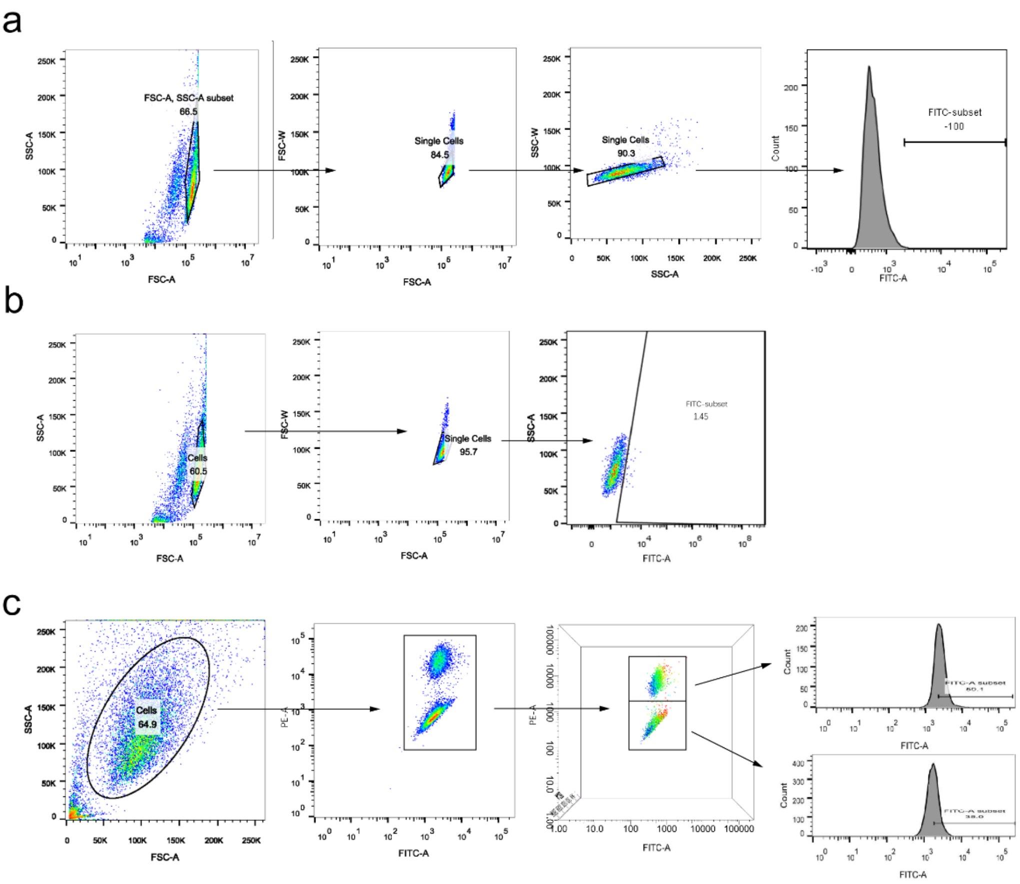

Supplementary Figure 16. Gating strategy for flow cytometric analysis. (a) Gating strategy for the flow cytometric analysis in Fig. 3f and Figure S6. (b) Gating strategy for the flow cytometric analysis in Fig. 4a. (c) Gating strategy for the flow cytometric analysis in Fig. 5b.

Supplementary Table 1. Sequences of oligonucleotides (red regions are the binding part of mRNA and staple).

Oligonucleotides	Sequence (5′-3′)
Splint 3-1	GGGUUUGGGUCAACUCUCCGGG
Splint 3-2	GGGCAGCUGCCCAGGCAGCGGG
RNA staple 3-1	GACCCAAACCCCCCCAUUGAAUGUCCUUCAGUCCCCCCGUUUAAACGCUAGCCAGCCCCCCGGAGAGUU
RNA staple 3-2	GGCAGCUGCCCCCCCAGUCCUACUUCCAGUCCCCCCCACCUGAAGCCUCCCAUCCCCCCGCUGCCUG
Splint 5-1	GGGAAACAUGUUAAAUAUUGGG
Splint 5-2	GGGGUCCACUGAAGGACAUUGG
RNA staple 5-1	ACAUGUUUCCCCCCGUUUAAACGCUAGCCAGCCCCCCCCAAUUCCAGCAGCAGCCCCCCCGACCCAAACAAAAGCGACCCCCCUAUCUGCAACAGUCCUUCCCCCCCAAUAUUUA
RNA staple 5-2	CAGUGGACCCCCCCAUCUUUUUUCUCCUUCACCCCCCUCAGUCUAAAGGUUGUGCCCCCCUUAUGAACAGCAUCUCCCCCCGGCCAGUAAUGUCCGGGCCCCCCAAUGUCCUU
Splint 7-1-1	GGACCCAAGACAGAGCAUCGGG
Splint 7-1-2	GGCUGGAAGUAGGACUGCAGGG
RNA staple 7-1-1	GUCUUGGGUCCCCCCACCUUUAUAUAUGCACUCCCCCCGGUCCACGUAUCCAUCACCCCCCUGCAGUCC
RNA staple 7-1-2	UACUUCCAGCCCCCCUCUGUCGAUGCACGAUUCCCCCCCAUGUUUUAGUUCAUUUCCCCCCUCCACCUUGUCUAUGGCCCCCCCGAUGCUCU
Splint 7-2-1	GGAGGAUCAGUAGGUGGAAGGG
RNA staple 7-2-1	ACUGAUCCUCCCCCCAUCACCUUCACCUUUACCCCCCCGAAAUGGGAGGCUGGAACCCCCCUAUGGCUGC
RNA staple 7-2-2	CCCCCAGUCCCCCCAGCAUUACUCUGCAGUGCCCCCCCAUUUACUAGGAUGAGCCCCCCCGUUUAAACGCUAGCCAGCCCCCCUUCCACCU
MYC F for Chip	TCGGTCCACAAGCTCTCCACTT
MYC R for Chip	CCTCCCACACGGAGTTCCCAAT

Supplementary Table 2. Antibodies used in this study.

Antibody	Catalog	Company	Detection	Dilution
Smad4	10231-1-AP	Proteintech	WB/Chip/IF	WB(1:1000)/ Chip(1:200)/ IF(1:500)
MYC	10828-1-AP	Proteintech	WB	1:1000
VEGFC	22601-1-AP	Proteintech	WB	1:1000
CXCL5	ab126763	Abcam	WB	1:1000
Integrin β3	18309-1-AP	Proteintech	WB	1:1000
β-actin	20536-1-AP	Proteintech	WB	1:10000
Histone H3	ab1791	Abcam	WB	1:5000
HRP-Goat Anti-Mouse IgG(H+L)	SA00001-1	Proteintech	WB	1:5000
HRP-Goat Anti-Rabbit IgG(H+L)	SA00001-2	Proteintech	WB	1:5000
FITC-Goat Anti-Rabbit IgG(H+L)	SA00003-2	Proteintech	IF	1:500
