# Supplementary material for: Lantern-shaped flexible RNA origami for Smad4 mRNA delivery and growth suppression of colorectal cancer
Source: Nat Commun. 2023 Mar 10;14:1307. doi: 10.1038/s41467-023-37020-y (PMC9998469; doi:10.1038/s41467-023-37020-y)
Supplement: Supplementary file 3 — Reporting Summary [file 41467_2023_37020_MOESM3_ESM.pdf]

nature portfolio

Corresponding author(s): Bujun Ge, Fenyong Sun, Xiaoli Zhu

Last updated by author(s): Feb 20, 2023

Reporting Summary

Nature Portfolio wishes to improve the reproducibility of the work that we publish. This form provides structure for consistency and transparency in reporting. For further information on Nature Portfolio policies, see our Editorial Policies and the Editorial Policy Checklist.

Statistics

For all statistical analyses, confirm that the following items are present in the figure legend, table legend, main text, or Methods section.

n/a	Confirmed	
☐	☒	The exact sample size (*n*) for each experimental group/condition, given as a discrete number and unit of measurement
☐	☒	A statement on whether measurements were taken from distinct samples or whether the same sample was measured repeatedly
☐	☒	The statistical test(s) used AND whether they are one- or two-sided<br>*Only common tests should be described solely by name; describe more complex techniques in the Methods section.*
☒	☐	A description of all covariates tested
☐	☒	A description of any assumptions or corrections, such as tests of normality and adjustment for multiple comparisons
☐	☒	A full description of the statistical parameters including central tendency (e.g. means) or other basic estimates (e.g. regression coefficient) AND variation (e.g. standard deviation) or associated estimates of uncertainty (e.g. confidence intervals)
☐	☒	For null hypothesis testing, the test statistic (e.g. *F*, *t*, *r*) with confidence intervals, effect sizes, degrees of freedom and *P* value noted<br>*Give P values as exact values whenever suitable.*
☒	☐	For Bayesian analysis, information on the choice of priors and Markov chain Monte Carlo settings
☒	☐	For hierarchical and complex designs, identification of the appropriate level for tests and full reporting of outcomes
☐	☒	Estimates of effect sizes (e.g. Cohen's *d*, Pearson's *r*), indicating how they were calculated

Our web collection on statistics for biologists contains articles on many of the points above.

Software and code

Policy information about availability of computer code

Data collection	No custom computer code or algorithm was used in this study.
Data analysis	Microsoft Excel 2021, Image J 1.47t, GraphPad Prism 8, FlowJo v10.6.2, SPSS 26.0, JASPAR 2016

For manuscripts utilizing custom algorithms or software that are central to the research but not yet described in published literature, software must be made available to editors and reviewers. We strongly encourage code deposition in a community repository (e.g. GitHub). See the Nature Portfolio guidelines for submitting code & software for further information.

Data

Policy information about availability of data

All manuscripts must include a data availability statement. This statement should provide the following information, where applicable:

- Accession codes, unique identifiers, or web links for publicly available datasets
- A description of any restrictions on data availability
- For clinical datasets or third party data, please ensure that the statement adheres to our policy

The potential recognition sites of Smad4 in the MYC promoter region was obtained from JASPAR database (http://jaspar2016.genereg.net). All data generated or analyzed during this work are included in this article and its Supplementary Information files. Source data are provided with this paper.

Human research participants

Policy information about studies involving human research participants and Sex and Gender in Research.

Reporting on sex and gender	N/A
Population characteristics	N/A
Recruitment	N/A
Ethics oversight	N/A

Note that full information on the approval of the study protocol must also be provided in the manuscript.

Field-specific reporting

Please select the one below that is the best fit for your research. If you are not sure, read the appropriate sections before making your selection.

[x] Life sciences [ ] Behavioural & social sciences [ ] Ecological, evolutionary & environmental sciences

For a reference copy of the document with all sections, see nature.com/documents/nr-reporting-summary-flat.pdf

Life sciences study design

All studies must disclose on these points even when the disclosure is negative.

Sample size	No statistical methods were used to determine the sample size. They were determined based on prior experience or pilot experiments. At least n=3 biologically independent repeats were performed per experimental condition in line with standards. The numbers of performed experiments were indicated in each figure legend.
Data exclusions	No data were excluded.
Replication	Replication attempts were successful. All experiments were conducted at least triplicates independently to guarantee reproducibility.
Randomization	Samples were randomly allocated in the study.
Blinding	No blinding was used in the study due to the complicated experimental design. But the investigators were blinded to sample collection and data analysis.

Reporting for specific materials, systems and methods

We require information from authors about some types of materials, experimental systems and methods used in many studies. Here, indicate whether each material, system or method listed is relevant to your study. If you are not sure if a list item applies to your research, read the appropriate section before selecting a response.

Materials & experimental systems

n/a	Involved in the study	
[ ]	[x]	Antibodies
[ ]	[x]	Eukaryotic cell lines
[x]	[ ]	Palaeontology and archaeology
[ ]	[x]	Animals and other organisms
[x]	[ ]	Clinical data
[x]	[ ]	Dual use research of concern

Methods

n/a	Involved in the study	
[x]	[ ]	ChIP-seq
[ ]	[x]	Flow cytometry
[x]	[ ]	MRI-based neuroimaging

Antibodies

Antibodies used	Primary antibody:<br>Smad4, Cat:10231-1-AP, Proteintech,WB(1:1000)/Chip(1:200)/IF(1:500)<br>Myc, Cat:10828-1-AP, Proteintech, WB(1:1000)<br>VEGFC, Cat:22601-1-AP, Proteintech, WB(1:1000)<br>CXCL5, Cat:ab126763, Abcam, WB(1:1000)

	Integrin β3, Cat:18309-1-AP, Proteintech, WB(1:1000)<br>β-actin, Cat:20536-1-AP, Proteintech, WB(1:10000)<br>Histone H3, Cat: ab1791, Abcam, WB(1:5000)<br>Secondary antibody:<br>HRP-Goat Anti-Mouse IgG(H+L), Cat:SA00001-1, Proteintech, WB (1:5000)<br>HRP-Goat Anti-Rabbit IgG(H+L), Cat:SA00001-2, Proteintech, WB (1:5000)<br>FITC-Goat Anti-Rabbit IgG(H+L), Cat:SA00003-2, Proteintech, IF(1:500)
Validation	Validation of the use of Smad4 antibody for WB/Chip/IF has been provided by the manufacture's website. https://www.ptgcn.com/products/SMAD4-Antibody-10231-1-AP.htm<br>Validation of the use of Myc antibody for WB has been provided by the manufacture's website. https://www.ptgcn.com/products/MYC-Antibody-10828-1-AP.htm<br>Validation of the use of VEGFC antibody for WB has been provided by the manufacture's website. https://www.ptgcn.com/products/VEGFC-Antibody-22601-1-AP.htm<br>Validation of the use of CXCL5 antibody for WB has been provided by the manufacture's website. https://www.abcam.cn/cxcl5-antibody-epr44502-ab126763.html<br>Validation of the use of Integrin β3 antibody for WB has been provided by the manufacture's website. https://www.ptgcn.com/products/ITGB3-Antibody-18309-1-AP.htm<br>Validation of the use of Histone H3 antibody for WB has been provided by the manufacture's website. https://www.abcam.cn/histone-h3-antibody-nuclear-marker-and-chip-grade-ab1791.html<br>Validation of the use of β-actin antibody for WB has been provided by the manufacture's website. https://www.ptgcn.com/products/ACTB-Antibody-20536-1-AP.htm<br>Validation of the use of HRP-Goat Anti-Mouse IgG(H+L) antibody for WB has been provided by the manufacture's website. https://www.ptgcn.com/products/HRP-conjugated-Affinipure-Goat-Anti-Mouse-IgG-H-L-secondary-antibody.htm<br>Validation of the use of HRP-Goat Anti-Rabbit IgG(H+L) antibody for WB has been provided by the manufacture's website. https://www.ptgcn.com/products/HRP-conjugated-Affinipure-Goat-Anti-Rabbit-IgG-H-L-secondary-antibody.htm<br>Validation of the use of FITC-Goat Anti-Rabbit IgG(H+L) antibody for IF has been provided by the manufacture's website. https://www.ptgcn.com/products/Fluorescein-FITC-conjugated-Affinipure-Goat-Anti-Rabbit-IgG-H-L-secondary-antibody.htm

Eukaryotic cell lines

Policy information about cell lines and Sex and Gender in Research

Cell line source(s)	The human CRC cell lines SW480 (CCL-228), SW620 (CCL-227), HT29 (HTB-38) were purchased from the American type culture collection (ATCC), human colonic epithelial cell (HCoEpiC, 2950) was obtained from ScienCell Research Laboratories.
Authentication	Cell lines were authenticated by morphological examination.
Mycoplasma contamination	Negative for mycoplasma.
Commonly misidentified lines<br>(See ICLAC register)	No commonly misidentified cell lines were used.

Animals and other research organisms

Policy information about studies involving animals; ARRIVE guidelines recommended for reporting animal research, and Sex and Gender in Research

Laboratory animals	Female BALB/c nude mice (Purchased from Shanghai SLAC Laboratory Animal Company, Shanghai, China) , 4-5 weeks, housed in a barrier facility on a 12 h light/dark cycle at 22-24°Cand 45-55% % humidity.
Wild animals	The study did not involve wild animals.
Reporting on sex	Only female nude mice was used in the study.
Field-collected samples	The study did not involve field-collected samples.
Ethics oversight	All experiments of animal were in accordance with the guidelines on the use and care of laboratory animals for biomedical research published by the National Institutes of Health, and approved by the committee on the Ethics of Animal Experiments of Shanghai University (No. 2022-238).

Note that full information on the approval of the study protocol must also be provided in the manuscript.

Flow Cytometry

Plots

Confirm that:

- [x] The axis labels state the marker and fluorochrome used (e.g. CD4-FITC).
- [x] The axis scales are clearly visible. Include numbers along axes only for bottom left plot of group (a 'group' is an analysis of identical markers).
- [x] All plots are contour plots with outliers or pseudocolor plots.
- [x] A numerical value for number of cells or percentage (with statistics) is provided.

Methodology

Sample preparation	Cells were cultured in the 12 well plates and treated with FITC labeled mRNA nano-lantern for different time points (0, 1, 2 h) followed by washing with PBS three times. The cells were collected for flow cytometry analysis.<br>For cell targeting experiments, the HCoEpiC and SW480 were co-incultured in a 12 well plates and treated with FITC labeled mRNA nano-lantern for 2 h. Then, the cells were harvested and probed with PE-Intergrin β3 antibody, which was employed to lable the Intergrin β3 positive CRC cells. The cells were washed with PBS three times and then collected for flow cytometry analysis. The percentage of positive cells was evaluated by Flowjo software.
Instrument	FACScelesta(BD)
Software	FlowJo (v10.6.2)
Cell population abundance	10000 cells were measured for analysis every time.
Gating strategy	The gating strategy was provided in the Supplementary Figure 16.

- [x] Tick this box to confirm that a figure exemplifying the gating strategy is provided in the Supplementary Information.
